# Clinical and Epidemiological Study on Tubercular Uveitis in a Tertiary Eye Care Centre in Italy

**DOI:** 10.1155/2020/4701820

**Published:** 2020-03-14

**Authors:** M. La Cava, A. Bruscolini, M. Sacchetti, M. P. Pirraglia, A. Moramarco, M. Marenco, G. Iaiani, G. Covelli, T. Rizzo, I. Abicca, A. Lambiase

**Affiliations:** ^1^Department of Sense Organs, Sapienza University of Rome, Rome, Italy; ^2^Department of Public Health and Infectious Diseases, Sapienza University of Rome, Rome, Italy

## Abstract

**Purpose:**

To describe frequency, clinical characteristics, and visual prognosis of tubercular uveitis (TBU) in a nonendemic country.

**Methods:**

We retrospectively reviewed 3743 charts of patients with endogenous uveitis visited from 2008 to 2018 at a tertiary referral centre in Rome, Italy. We included immunocompetent patients with diagnosis of TBU. Patients were divided in two groups: patients with history of uveitis without a previous diagnosis of TBU (group A) and patients at their first episode of TB uveitis (group B).

**Results:**

TBU was diagnosed in 28 (0.75%) out of 3743 patients. Twelve (42.9%) patients came from tuberculosis endemic areas. All patients received specific antitubercular treatment (ATT) and were evaluated for a mean follow-up of 3.2 ± 2.9 years. Group A showed a greater number of ocular complications when compared with group B. ATT was effective in reducing the frequency of recurrences of uveitis in patients of group B.

**Conclusion:**

Intraocular inflammation can be the first manifestation of tuberculosis. Our data highlight that early diagnosis and specific treatment of TBU may allow to decrease recurrences and to improve visual outcomes.

## 1. Introduction

Tuberculosis (TB) is an infective disease caused by Mycobacterium tuberculosis (MT) or by other members of the MT complex, and it represents one of the main causes of mortality and morbidity in the world [1]. Tuberculosis is characterized by pulmonary and extrapulmonary manifestations which may involve the skin, eye, and nervous, cardiovascular, gastrointestinal, or genitourinary systems [2]. The main manifestation of ocular TB is uveitis. The prevalence of TB uveitis (TBU) is estimated between 9% and 11% in endemic countries and between 1% and 6% in nonendemic countries [3–6]. In Italy, the frequency of TBU is estimated to be approximately 7% [7, 8].

Intraocular TB is rare in active lung TB, while it is more frequent in patients with advanced tubercular lesions or extrapulmonary forms [9]. Trauma, immunosuppression, and malnutrition are predisposing factors for development of intraocular TB [10]. The pathogenesis of ocular involvement in TB is controversial. It has been supposed that TBU may result from direct mycobacterium infection and/or from a delayed hypersensitivity reaction to mycobacterial antigens [11].

The diagnosis and management of TBU represent a challenge for ophthalmologists, due to the high variability of clinical manifestations, poor associated systemic signs and lack of consensus on specific treatment. Specifically, results are controversial about the specific anti-TB treatment (ATT) in patients with TBU [6, 12]. The most frequent ATT for patients with TBU is the use of four drugs (Isoniazid, Rifampicin, Ethambutol, and Pyrazinamide) for 2 months followed by two drugs, Rifampicin and Isoniazid, for 4 months [13–15]. In addition, patients with TBU may develop ocular complications which require specific treatments, such as corticosteroids for cystoid macular edema (CME) or ocular surgery in patients with cataract [16–18].

Few data are available on clinical presentation, management, and outcomes of TBU in nonendemic countries, including Italy. Cimino et al. described the course and clinical outcomes of TBU in an Italian population and reported that specific ATT was effective in reducing the number of uveitis relapses and to improve visual outcome [19].

Currently, no data are available on the effect of early ATT on long-term clinical outcomes in patients with TBU. Therefore, the aim of this study is to describe clinical and epidemiological characteristics of patients with TBU referred at a tertiary eye centre in Rome, Italy, in order to evaluate the effects of an early diagnosis and specific ATT on visual outcomes and recurrence rate.

## 2. Materials and Methods

The clinical charts of all patients referred at a tertiary referral uveitis centre, the Ocular Immunovirology Service, at Sapienza University of Rome, from January 1st, 2008, to December 31st, 2018, were retrospectively reviewed. The study was performed in accordance with the tenets of the Declaration of Helsinki, and Institutional Review Board approval was obtained.

The inclusion criteria were as follows:

1. Diagnosis of uveitis, with or without involvement of the optic nerve
2. Positive tuberculin skin test (TST) (>10 mm at 72 h) and, in doubtful cases, positive interferon gamma release assay (IGRA), including the QuantiFERON-TB Gold.
3. Specific treatment with antituberculosis treatment during follow-up

We excluded patients with other syndromes or infections or acquired immunodeficiency syndrome and patients in which diagnosis of TB was excluded based on results of clinical evaluation, TST, IGRA tests, and chest imaging.

Diagnosis of TBU was performed by a uveitis specialist, based on clinical history and complete eye examination, and supported by diagnostic exams, including TST and IGRA tests and chest radiography or contrast-enhanced computed tomography (CT) scan.

Ocular and systemic clinical history and results of complete eye exam were recorded, including visual acuity assessment by Snellen charts, slit lamp examination, intraocular pressure (IOP) values assessed by Goldman applanation tonometer, and fundus examination. In posterior and diffuse uveitis, retinal vascularization was evaluated by Fluorangiography (FA), and macular morphology was studied by Spectral Domain Optical Coherence Tomography (SD-OCT, Heidelberg Spectralis, Heidelberg Engineering, Heidelberg, Germany).

Uveitis was classified as anterior, intermediate, posterior, and diffuse uveitis, in accordance with the International Uveitis Study Group diagnostic criteria [20].

Clinical course, recurrence rate of uveal inflammation, ocular complications, and visual acuity were evaluated during the entire follow-up period. All topical and systemic therapies, side effects of systemic treatments, and ocular surgery performed during the follow-up period were also collected.

To evaluate the effects of early diagnosis and specific ATT on clinical and visual outcomes, patients were divided in 2 groups based on their clinical history at the first visit in our centre. Specifically, patients with diagnosis of uveitis from at least 1 year, without previous etiological diagnosis of TBU and not treated with specific ATT, were included in group A. Patients at their first episode of uveitis, with a diagnosis of TBU treated with ATT at the onset, were included in group B.

The following ocular parameters were compared between groups: (i) visual acuity, (ii) number of ocular recurrences of TBU, and (iii) ocular complications.

The exact Fisher test and independent *t*-test were used to compare data in between groups. The paired samples *T*-test was used to evaluate changes in BCVA within groups. *P* values < 0.05 were considered statistically significant (SPSS version 24).

## 3. Results

In this retrospective study were included 28 (0.75%) patients with TBU out of 3743 patients with uveitis visited in our center, from 2008 to 2018. The mean age of patients with TBU was 48.8 ± 16.3 years (range 14–80 years). Sixteen were females (57.1%) and 12 males (42.9%). Twelve (43%) out of 28 patients came from endemic countries for TB, and 16 patients (57%) were born in Italy. Anterior uveitis was the most frequent form of uveitis, observed in 12 patients (43%), 2 of which were granulomatous uveitis. Diffuse uveitis was observed in 9 patients, 4 of which were granulomatous, while 7 showed a posterior uveitis (Table 1). Sixteen uveitis were bilateral (57%) and 12 monolateral (43%). Among the patients with posterior uveitis, 5 (71%) were Caucasian white and 2 (29%) were Asian. Demographic and clinical characteristics of patients included in the study are described in Table 1.

All patients showed positive (>10 mm after 72 h) TST. The QuantiFERON-TB-Gold test was performed in 11 patients and resulted positive in all cases. A chest radiography was performed in all patients and showed an active pulmonary lesion in 1 patient (4%), no visible pulmonary lesions in 13 (46%), and lung calcific nodules in 14 (50%) cases. Ten patients (36%) underwent additional chest CT scan, showing the presence of lung lesions in 8 cases, with an active lesion in 1 patient.

Twelve patients (20 eyes) with TBU were referred to our centre with a diagnosis of uveitis of unknown aetiology in the previous 1–39 years (mean 11.8 ± 12.7 years). Sixteen patients (24 eyes) at their first episode of TBU were included in group B.

**Figure 1:**
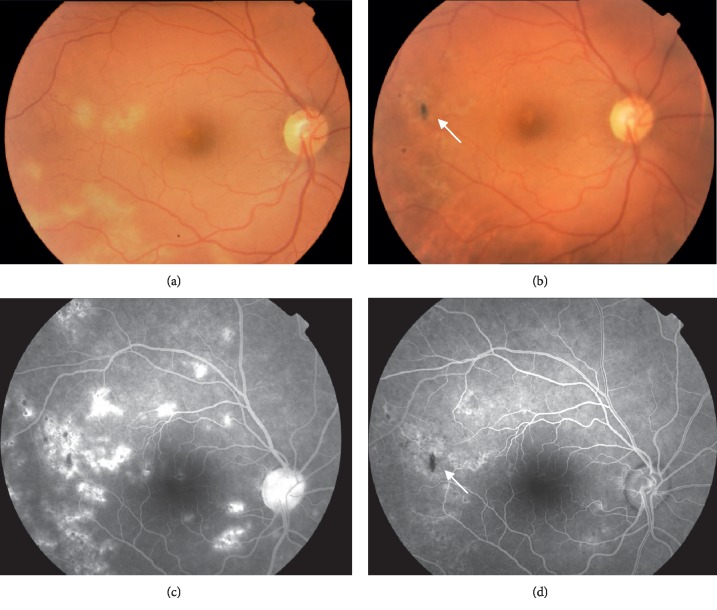
Ophthalmoscopic and fluorescein angiographic (FA) images of a patient with posterior tubercular uveitis. Before treatment, fundus photograph (a) and FA (b) show multiple cream-yellow active lesions with increase of choroidal fluorescence at FA. Twelve months after antitubercular treatment, fundus photograph (c) and FA (d) show fading inactive lesions and mottling pigment (white arrow) with decrease of choroidal fluorescence.

Seven (58%) patients included in group A showed anterior uveitis, 1 showed posterior uveitis, and 4 (33%) showed diffuse uveitis. In the patients at their first episode of TBU (group B), we observed posterior uveitis in 38% of cases, followed by anterior (31%) and diffuse uveitis (31%) (Figure 1).

Eighteen (64%) out of 28 patients received standard fourfold ATT for TB, including Isoniazid 5 mg/kg/die, Rifampicin 600 mg/die, Ethambutol 15 mg/kg/die, and Pyrazinamide 25–30 mg/kg/die. Seven patients were treated only with Isoniazid 5 mg/Kg/die. Rifampicin 600 mg/die was prescribed in one case of panniculitis. Two patients were treated for MDR forms. Because of high toxicity of anti-TB drugs, liver and kidney function were regularly monitored with periodic blood exams. A slight increase of hepatic function indexes was observed in 1 patient during TB therapy, which did not require ATT discontinuation. Side effects of ATT included 1 optic neuritis due to treatment with Ethambutol, 1 gastric intolerance which resulted in the replacement of standard ATT with Moxifloxacin and Ethambutol, and 1 case of anaemia, treated with oral iron supplementation. Two patients reported skin rashes and were treated with systemic antihistamines. The characteristics of specific treatment in the two groups are described in Table 2. During the follow-up period, patients underwent periodic eye examinations in our centre.

Patients with posterior uveitis were treated with oral corticosteroids (prednisolone 1 mg/kg) associated with periocular corticosteroids injections (methylprednisolone 40 mg/ml) in 2 cases. Patients with diffuse uveitis received an association of mydriatic eye drops and local and systemic corticosteroids.

Patients were periodically evaluated during ATT, and at the end of treatment, in order to evaluate ocular complications and TBU relapses. The mean follow-up was 3.2 ± 2.9 years (range: 1–11.3 years), 4.2 ± 3.1 years in group A, and 2.3 ± 2.6 years in group B. Ocular complications of TBU were observed in 10 patients, 4 (25%) in group A, and 6 (60%) in group B (Table 1).

Cystoid macular edema was treated with oral acetazolamide and oral steroids. All patients with cataract underwent cataract surgery. Glaucoma was treated with topical medical therapy in 2 patients. Only one patient required surgical iridectomy in addition to topical medications for glaucoma.

**Figure 2:**
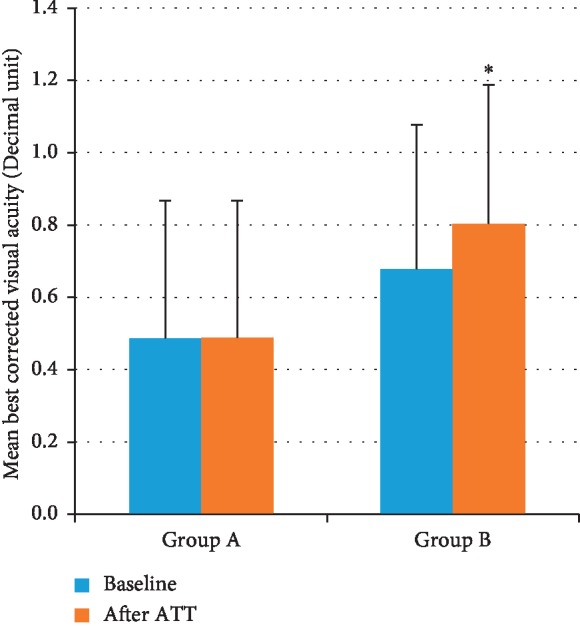
Mean best corrected visual acuity at baseline and after antitubercular treatment in group A and B.

Mean best correct visual acuity (BCVA) before ATT was not different between the two groups (group A: 0.5 ± 0.38; group B: 0.67 ± 0.4 decimal units). After specific ATT, group B showed a significant improvement of mean BCVA (group B: mean BCVA: 0.8 ± 0.38 *p*=0.016 vs. baseline), while patients in group A did not show a significant improvement of BCVA (group A mean BCVA after ATT 0.48 ± 0.38). In addition, improvement of BCVA was significantly higher in group B (mean BCVA change from baseline 0.12 ± 0.23 decimal units), when compared with group A (mean BCVA change from baseline −0.03 ± 0.21; *p*=0.024 decimal units) (Figure 2).

After ATT, the number of recurrences of the uveitis in group A was significantly lower (mean 0.35 ± 2 recurrences/year) when compared with the period before the diagnosis of TBU with ATT (mean 1.94 ± 2 recurrences/year; *p* > 0.001). During follow-up, after ATT, 5 (42%) patients in Group A showed recurrence of TBU and only 1 patient in group B (6%).

## 4. Discussion

This retrospective study showed that the proportion of TB uveitis among patients referred to our tertiary eye care centre from 2008 to 2018 was 0.75%. The frequency of TBU in our center is decreased when compared with the previous studies (1.76% in the period between 1986 and 1999 and 1.74% between 1999 and 2003) [21]. This slight decrease of incidence of TBU may reflect the trend of incidence of the disease, and alternatively, it may be explained by an improvement of diagnostic techniques, which allows to correctly diagnose uveitis associated with sarcoidosis, histoplasmosis, and other autoimmune diseases, that, in the past, were classified as TBU [22, 23]. Our diagnosis of TBU was based on the presence of clinical signs of uveitis associated with positive TST (>10 mm at 72 h) and/or IGRA tests and exclusion of other causes of uveitis. There is a lack of consensus on the diagnostic criteria of TBU. Gupta et al. proposed a classification of intraocular TB (IOTB) as “confirmed,” “probable,” or “possible” [24]. They defined “confirmed IOTB” ocular inflammation associated with a positive microbiological test for Mycobacterium tuberculosis in ocular fluids (aqueous humor or vitreous) or tissues [24]. However, vitreous and ocular tissues are difficult to collect, and microbiological test for *Mycobacterium tuberculosis* in aqueous humor are frequently false negative. “Probable IOTB” was defined in cases in which uveitis is associated with pulmonary or extrapulmonary active TB, positive chest X-ray or immunologic tests for TB, or response to ATT, and other aetiologies of uveitis are excluded [24]. An intraocular inflammation is considered “possible IOTB” in the presence of ocular signs suggestive of TBU, associated with positive TST or IGRA, without pulmonary or extraocular signs of TB infection. According to the Tuberculosis Committee of the Portuguese Society of Pulmonology and of Ophthalmology, diagnosis of ocular TB could be performed in the presence of ocular findings suggesting ocular TB associated with TST or IGRA positivity (≥10 mm in immunocompetent patients) or chest radiology signs of TB [25]. Recently, a group of 25 ophthalmic centres analysed several clinical features in a larger cohort of TBU cases in order to identify novel and more standardized management protocols [26].

In our study, 42.9% of patients came from endemic areas, of which 91.7% were under 65 years of age. Conversely, 57.1% of our population was born in Italy, with 68.8% under 65 years of age. This finding is in line with the epidemiological evidence of an earlier onset of the disease in endemic areas [27].

In the population of this study, we observed significant differences neither in the type of uveal involvement by TB, in the granulomatous and nongranulomatous forms, nor in the monolateral/bilateral presentation. By comparing patients in group A and B, we observed that patients of group A showed higher frequency of anterior uveitis, while no differences in anatomical sites was observed in group B. This finding may suggest that tubercular aetiology should be investigated also in patients with anterior uveitis.

After diagnosis and specific treatment with ATT, we observed an improvement of symptoms and a reduction of frequency of relapses. We treated most of the patients with TBU with four drugs (Isoniazid, Rifampicin, Ethambutol, and Pyrazinamide) for 2 months and subsequently with two drugs, Rifampicin and Isoniazid, for 4 months. In line with previous studies, the most frequent complication in our study was CME [28].

The necessity to treat TB uveitis with ATT is controversial. Previous studies suggested that ATT treatment positively influences ocular relapses, inducing a remission of the disease in a higher number of patients, when compared with untreated group (84% and 58% of patients, respectively) [2, 26]. In line with these studies, in our population, 78% of patients did not show relapses of the uveitis after ATT during the entire follow-up period [2, 26].

La Distia Nora et al. reported that that ATT does not influence visual prognosis after 1 year of follow-up in a nonendemic country. However, this study evaluated all ocular inflammation associated with the positive Quantiferon-TB Gold test, including uveitis and scleritis, in both immunocompetent and immuno-deficient patients [29]. Brunner et al. also evaluated patients affected by retinal vasculitis and positive TB gold in a nonendemic country and concluded that fourfold treatment did not influence the extension of vasculitis nor visual acuity, but was effective in reducing recurrences of ocular inflammation [30]. A retrospective study in a uveitis centre of Birmingham also reported that 6 months after ATT, 90% of patients were flare free, and 80% were flare free at 12 months. The authors also reported that patients treated with ATT within 8 months of ocular onset did not show significant difference in the time to flare-up when compared with patients untreated for a longer time [31]. In contrast, our data showed lower recurrence rate in the group of patients which received an early treatment. Indeed, we divided our population in two groups: group A, including patients in which the etiological diagnosis of TBU was not performed for long time and group B, in which patients received a diagnosis of TBU at their first episode of uveitis. Furthermore, we have evaluated the difference in ocular recurrences and in visual outcomes between the two groups, before and after ATT. We observed that patients in Group B, treated with ATT at their first episode of TBU, showed a higher improvement of visual acuity, highlighting the importance of a prompt diagnosis and an early specific treatment. This finding is in line with previous studies reporting the efficacy of ATT in improving visual outcome [27, 32]. In addition, after ATT, patients in group B showed a lower number of recurrences during the follow-up when compared with group A. Regarding ATT tolerability, only 5 patients had some side effects, which did not require discontinuation of treatment.

## 5. Conclusion

In conclusion, in this study, we demonstrate that early diagnosis and specific treatment for TBU allowed a decrease of recurrences and a significant improvement of visual acuity. Our results also highlight the importance of considering TB as a possible cause of idiopathic uveitis, also in nonendemic countries.

## Figures and Tables

**Table 1:** Demographic characteristics, uveitis features, and ocular complications of the patients included in this study.

Variable	All patients	Group A	Group B
Patients (n)	28	12	16

Male (n; %)	12 (42.9%)	5 (41.7%)	7 (43.7%)

Female (n, %)	16 (57.1%)	7 (58.3%)	9 (56.2%)

Age (mean ± SD)	48.79 ± 16.26	58.83 ± 11.7	41.25 ± 15.31

Country of origin			
Bangladesh	3		3
Bulgaria	1		1
Egypt	1	1	
India	1	1	
Italy	**16**	**10**	**6**
Morocco	1		1
Moldavia	1		1
Nigeria	1		1
Pakistan	1		1
Romania	2		2

Monolateral uveitis (n; %)	12 (43%)	4 (33%)	8 (50%)

Bilateral uveitis (n; %)	16 (57%)	8 (66%)	8 (50%)

Anterior uveitis (n; %)	12 (43%)	7 (58%)	5 (31%)

Posterior uveitis (n; %)	7 (25%)	1 (9%)	6 (38%)

Diffuseuveitis (n; %)	9 (32.1%)	4 (33%)	5 (31%)

TBU complications (n; %)	10 (36%)	4 (25%)	6 (60%)
CME (n)	3	3	
Cataract (n)	2		2
Glaucoma (n)	1	1	
CME and cataract (n)	2		2
CME and glaucoma (n)	1		1
Glaucoma and cataract (n)	1		1

n: number; SD: standard deviation; TBU: tubercular uveitis, CME: cystoid macular edema.

**Table 2:** Antitubercular treatment in the two groups. (PYR = Pyrazinamide; ETHA = Ethambutol; RIF = Rifampicin; ISO = Isoniazid; and CLA = Clarithromycin).

Patient	Therapy	Duration	Side effects
Group A			
1	ISO	6 months	—
2	PYR ETHA RIF ISO followed by RIF ISO	2 months 4 months	—
3	RIF	3 months	—
4	ISO	2 months	—
5	ISO	12 months	—
6	PYR ETHA RIF ISO followed by RIF ISO	0.5 month 1 month	Gastric intolerance
7	PYR ETHA RIF ISO followed by RIF ISO	2 months 3 months	—
8	PYR ETHA RIF ISO followed by RIF ISO	2 months 4 months	—
9	PYR ETHA RIF ISO followed by RIF ISO	2 months 4 months	Increase of blood liver enzymes
10	PYR ETHA RIF ISO followed by RIF ISO	2 months 4 months	—
11	ISO	4 months	—
12	ISO	8 months	—

Group B			
13	PYR ETHA RIF ISO followed by RIF ISO	2 months 4 months	—
14	PYR ETHA RIF ISO	Not compliant	—
15	PYR ETHA RIF ISO followed by RIF ISO	2 months 4 months	—
16	PYR ETHA RIF ISO followed by RIF ISO	2 months 4 months	Optic neuritis
17	RIF ETHA AZT	12 months	—
18	CLA ETHA	12 months	—
19	PYR ETHA RIF ISO followed by RIF ISO	2 months 4 months	Itching
20	PYR ETHA RIF ISO followed by RIF ISO	2 months 4 months	—
21	ISO	5 months	—
22	PYR ETHA RIF ISO followed by RIF ISO	2 months 4 months	—
23	ISO	Not compliant	—
24	PYR ETHA RIF ISO followed by RIF ISO	2 months 4 months	—
25	PYR ETHA RIF ISO followed by RIF ISO	2 months 4 months	Itching
26	PYR ETHA RIF ISO followed by RIF ISO	2 months 4 months	Anemy
27	PYR ETHA RIF ISO followed by RIF ISO	2 months 2 months	—
28	PYR ETHA RIF ISO	1 month	—

Patients with anterior uveitis received topical ophthalmic treatment with mydriatic eye drops associated with topical dexamethasone 0.2% eye drops.

## Data Availability

The clinical and epidemiological data used to support the findings of this study are available from the corresponding author upon request.
